# The outcomes of blastocyst versus cleavage stage embryo biopsy for preimplantation genetic testing for monogenic diseases

**DOI:** 10.3389/fendo.2025.1518760

**Published:** 2025-04-03

**Authors:** Lilach Marom Haham, Adva Aizer, Almog Arad, Jigal Haas, Oshrit Lebovitz, Eran Zilberberg, Ravit Nahum, Raoul Orvieto

**Affiliations:** ^1^ Department of Obstetrics and Gynecology, Chaim Sheba Medical Center, Ramat Gan, Israel; ^2^ Sackler Faculty of Medicine, Tel-Aviv University, Tel Aviv, Israel; ^3^ The Tarnesby-Tarnowski Chair for Family Planning and Fertility Regulation, Sackler Faculty of Medicine, Tel-Aviv University, Tel Aviv, Israel

**Keywords:** preimplantation genetic testing for monogenic disease (PGT-M), Trophectoderm biopsy, blastomere biopsy, implantation potential, reproductive outcomes

## Abstract

In recent years, the application of blastocyst biopsy in PGT has been gradually rising, mainly due to the assumed detrimental effect of blastomere biopsy on the embryo implantation potential and the widespread application of PGT for aneuploidy. In contrast to complete chromosomal testing (CCT) cycles, for which trophectoderm (TE) biopsy has become the well-established preferred method due to higher diagnostic reliability, evidences for the purpose of PGT-M are still lacking. Therefore, we conducted a retrospective cohort study including 147 PGT-M cycles with at least eight high quality embryos (HQE) suitable for biopsy at the cleavage stage, 83 and 64 in the blastocyst and cleavage stage biopsy groups, respectively. Our results showed no significant differences in implantation rates (32.8% vs. 33.6%, p=0.9), clinical pregnancy rates (CPR) per transfer (30.3% vs. 33.0%, p=0.7), as well as cumulative CPR (46.2% vs. 38.3%, p=0.4). This study is the largest so far, demonstrating that blastocyst biopsy has higher cost-effectiveness over cleavage stage biopsy in good prognosis patient population. Moreover, our data is the first to show that blastomere biopsy does not compromise the reproductive outcomes, which merits further investigation regarding its cost-effectiveness in the poor prognosis patient population, having a small number of embryos for biopsy and transfer. Further large prospective randomized studies are needed to elucidate the preferred biopsy strategy in specific patient populations in order to provide a tailored treatment that will ensure the best prognosis for each patient.

## Introduction

Preimplantation genetic testing for monogenic diseases (PGT-M) is considered an alternative to prenatal diagnosis, enabling couples who are carriers of a genetic disease to select unaffected embryos for transfer, and deliver a healthy child (1). Since the first successful clinical application of blastomere biopsy and preimplantation genetic diagnosis of X chromosome linked diseases using PCR based methods for sex selection in 1990 (2), PGT-M has become widely adopted for testing various single gene diseases amongst are Huntington, Neurofibromatosis, Cystic fibrosis, Beta thalassemia, Fragile X syndrome, Duchenne muscular dystrophy etc. In fact, over 1000 genetic diseases are currently approved for PGT-M by the Human Embryology and Fertilization Authority (HEFA). Embryonic DNA sample for preimplantation testing can be achieved by three main approaches: [a] 1^st^ or 2^nd^ polar body (PB) biopsy from the mature oocyte and/or the zygote respectively; [b] blastomere biopsy at the cleavage stage, and [c] trophectoderm (TE) biopsy at the blastocyst stage (3). Over the years, the application of PB biopsy has been gradually reduced in favor of blastomere and TE biopsies due to the absence of well-established supporting data and the possibility of diagnostic inaccuracy as well as failure (1, 3) (4). According to the recent published European Society of Human Reproduction and Embryology (ESHRE) consortium data collection XXI, there is a reduction in the application of cleavage stage biopsy and concurrent increase in blastocyst biopsy for PGT-M (from 19% in 2016-1017 to 33% in 2018) (5), with blastocyst biopsy being performed in about half of the cases and in about the totality of the cases when associated with aneuploidy testing according to data analysis of 2020 presented in the last ESHRE congress. The improvement in extended culture and freezing methods, as well as evidence from studies indicating higher implantation and pregnancy rates per transfer following blastocysts compared to cleavage stage embryos (6, 7), are among the contributors to the shift in trend towards blastocyst culture, biopsy and transfer. Evidently, each biopsy strategy has its strengths and limitations. The main proposed advantages of blastocyst biopsy are (a) removal of smaller portion of the embryo as well as cells that are destined to become the placenta, which might be less detrimental for the embryo implantation potential, as opposed to removing a single blastomere which is a part of the embryo proper (8, 9). (b) Retrieving more cells with their corresponding DNA for analysis, which might result in higher sensitivity and reduced occurrences of failed diagnoses (3, 10). (c) Sampling and testing of embryos at the blastocyst stage having higher implantation potential, reduces the number of unnecessary tests due to the documented attrition between the cleavage and the blastocyst stage (10). However, the argued advantages of cleavage stage biopsy comprise of (a) providing the possibility of a fresh embryo transfer as opposed to blastocyst sampling which is bound to freezing after sampling and transferring the embryo at subsequent cycles. (b) The mechanism responsible to embryos arrest *in vitro* is not embryo aneuploidy, but rather other, such as culture conditions (11). Therefore, cleavage-stage embryo transfer might reduce the incidence of cycle cancellation due to failure of embryo development to the blastocyst stage and will provide the best cumulative live birth-rate per started cycle (12, 13). Moreover, cleavage stage transfer practice may result in improved pregnancy outcomes, in specific patient populations, as was recently demonstrated (14).

Studies comparing the clinical outcomes of cleavage stage to blastocyst biopsy for PGT-M are lacking (15). Only one study so far, by Kokkali et al, evaluated the implantation and clinical pregnancy rates between the two biopsy strategies in fresh transfer cycles. This study reported of lower implantation rates while performing cleavage stage biopsy and blastocyst transfer compared to blastocyst biopsy and transfer (16). However, its main limitation is the small sample size of each group which precludes the possibility to deduce solid conclusions regarding the preferred strategy.

Nowadays clinical practice aims to provide a personalized treatment according to patient’s characteristics, taking into consideration that “one size doesn’t fit all”. While the good prognosis patient population undergoing PGT-M has multiple embryos for biopsy and a fair chance for having a genetically normal embryo for transfer, the poor prognosis patient population faces a small number of embryos for biopsy either at the cleavage or the blastocyst stage with a higher risk for having no embryos for biopsy or transfer. The recent study by Xiao et al. which demonstrated significantly higher clinical pregnancy and live birth rates in transferring cleavage stage embryos compared to growing the embryos on and aiming for day 4-6 embryo transfer (14), gives rise to a need for providing additional evidence regarding the effect of cleavage stage compared to blastocyst biopsy in PGT-M on the reproductive outcomes. This evidence will assist in determining the preferred timing of the biopsy, for specific patients’ populations, maximizing treatment success.

In view of the aforementioned, we set out to evaluate the frozen embryo transfer outcomes of cleavage stage compared to blastocyst stage embryo biopsy in PGT-M cycles.

## Materials and methods

### Study design

This is a retrospective cohort study including patients admitted to the PGT-M program at the Sheba medical center’s IVF unit between January 2019 and December 2021. The study was approved by the institutional review board (#IRB SMC-9146-22).

### Patients’ eligibility criteria

Eligible patients were considered all consecutive women between 18- and 45-years old undergoing IVF-PGT-M cycles based on multiplex PCR programs designed for haplotyping using informative microsatellites markers (17). We included all cycles fulfilling the following criteria [a] with at least 8 high quality embryos (HQE) suitable for biopsy at the cleavage stage [b] subjected to freezing all embryos at the day of biopsy. We chose to include only cycles with at least 8 HQE at the cleavage stage because they are usually high responders subjected to freeze all, also in the cleavage stage biopsy group, allowing for a homogenous patient population in both study groups. HQE suitable for biopsy were defined as cleavage stage embryos with either 6 blastomeres and < 10% fragmentation or ≥ 7 blastomeres and < 15% fragmentation or reaching the morula stage. The decision on the day of biopsy was determined according to physician’s and embryologist’s discretion, as well as, patient’s desire. Women who are carriers of structural chromosomal anomalies undergoing PGT for structural rearrangement (PGT-SR) or patients undergoing PGT for aneuploidy (PGT-A) were excluded.

### Ovarian stimulation protocol, triggering and oocyte retrieval

Either the conventional GnRH antagonist or GnRH agonist/antagonist protocols were employed for ovarian stimulation (OS), as previously described (18) using various doses of gonadotropins. All cycles included medications with FSH (Follicle Stimulating Hormone, recombinant and/or urinary) and LH (Luteinizing Hormone, recombinant or human menopausal gonadotropin) activity. The specific protocol as well as the gonadotropin dose used in each cycle were left to the judgment of the treating physician based on pre-stimulation ovarian reserve measures (AFC, baseline FSH), female age and previous response to OS, if any. Final oocyte maturation was triggered at the presence of at least two leading follicles with a diameter of 18mm or greater by ultrasound assessment using either GnRH agonist (Decapeptyl 0.2mg) alone or a combination of GnRH agonist and human chorionic gonadotropin (hCG 250micrograms) (Dual trigger). Oocyte retrieval was carried out 36-38 hours post trigger under transvaginal ultrasound guidance.

Frozen embryo transfer was performed using either natural or artificial hormone replacement cycle based on the treating physician’s decision. Endometrial preparation and embryo transfer procedures used are described elsewhere (19). According to the practice in our IVF unit, un-affected frozen biopsied cleavage stage embryos were thawed at subsequent cycles, cultured overnight and were transferred at the morula or blastocyst stage. Un-affected blastocysts were thawed two hours prior to the scheduled time of the transfer.

### PGT-M procedure and molecular diagnosis

Cleavage stage embryos underwent blastomere biopsy at Day-3/4 as previously described (17, 20). Briefly, Day-3 embryos underwent blastomere biopsy using a micromanipulation system (Narashige, Japan) fitted on an inverted microscope (Diaphot 300, Nikon, Japan). A laser system (ZILOS-tk, Hamilton Thorne) was used for dissection of the zona pellucida prior to biopsy. A single blastomere was removed from each embryo and evaluated under ×400 inverted microscope for its integrity, presence of a nucleus, and being free from other cells\debris. Each blastomere was routinely washed in three drops of clean biopsy medium, prior to its transfer to the PCR tube, ensuring a pure sample. Day-4 embryos were placed in calcium magnesium free media (SAGE, CooperSurgical) for several minutes until they were de-compacted. Thereafter, the biopsy technique was performed as described for Day-3 embryo biopsy. Blastocyst stage biopsy was performed as follows: cleavage stage embryos destined to extended culture underwent laser assisted zona pellucida (ZP) opening on day 3. Upon reaching the expanded blastocyst stage, 5-7 herniated TE cells, opposite to the ICM, were removed using the standard flicking approach (21).

Re-biopsy was performed as follows: Day-4 embryos were placed in G-PGD TM media (Vitrolife) for 15 minutes until they were de-compacted and then the biopsy technique was performed as described for Day-3 embryos. Blastocysts were thawed 4-6 hours prior to re-biopsy, allowing time for re-expansion. Afterwards, the biopsy technique was performed as was described above for blastocyst stage embryos.

Molecular diagnoses classification of each embryo within the study groups included: [a] Complete diagnosis–unaffected or affected embryo according to the genetic disorder examined; [b] Incomplete diagnosis—suspected allele dropout or recombination; [c] PCR failure–no DNA is available for diagnosis; [d] Abnormal–the embryo has abnormal assembly of alleles–i.e. any structure different from one maternal and one paternal alleles matching the known haplotype, e.g. trisomy, monosomy or uniparental disomy.

### Study variables

The primary outcomes included the rate of complete diagnosis per ovum pick up (OPU), implantation rate (IR), clinical pregnancy rate (CPR) per transfer and cumulative clinical pregnancy rate per OPU. The IR was calculated by dividing the number of intrauterine gestational sacs observed on ultrasound by the number of embryos transferred. Clinical pregnancy was defined as an intrauterine gestation with a demonstration of fetal cardiac activity by ultrasound examination. Cumulative CPR per OPU was defined as the number of clinical pregnancies from one initiated cycle including all cycles of frozen embryos transferred, until one clinical pregnancy was achieved or all transferable embryos were used.

The secondary outcome was miscarriage rate (MR) defined as the proportion of spontaneous loss of an intra-uterine pregnancy (Gestational sacs observed by ultrasound examination).

Cycles’ missing data on the outcome measures were excluded from the study. BMI data were missing in 5 and 3 cases of the cleavage stage and blastocyst stage biopsy groups, respectively. Baseline E2 and FSH were missing in 11 and 10 cases of the cleavage and blastocyst stage biopsy groups respectively. Most of the missing data were E2 level at triggering, 61% and 49% of the cleavage stage and blastocyst stage biopsy groups, respectively. In those cases, we had data on the level at the day before the triggering. However, according to the recent ESHRE guideline, the addition of estradiol level measurements to ultrasound monitoring during ovarian stimulation is probably not recommended (22). Moreover, the data on the number of oocytes retrieved, which is a good measure of the ovarian response, is complete. Those parameters indicated as missing were removed from that specific analysis.

### Statistical analysis

Continuous variables are presented as mean ± standard deviation (SD) or median with interquartile range (IQR) according to distribution. Distributions were examined through assessment of skew and kurtosis. When these were found to be within the acceptable range, a mean was presented. Categorical variables are presented as counts/frequencies and percentages. Continuous variables were compared using Student’s t-test when data were normally distributed, and by Mann-Whitney U when the data were not normally distributed. Chi-squared tests were used to compare proportions. A p-value of 0.05 was used as a cutoff for statistical significance.

## Results

One hundred and thirty-four patients met the inclusion criteria in our PGT-M program between January 2019 to December 2021: sixty-one patients underwent 64 stimulation cycles performing cleavage stage biopsy and seventy-three patients underwent 83 stimulation cycles performing blastocyst biopsy. The most common indication for PGT-M was autosomal dominant diseases (46.9%), followed by X- linked (X-linked recessive, 16.3%; X-linked dominant, 11.6%) and autosomal recessive diseases (18.4%). Testing for multigenic mutations accounted for 6.8% of our study cohort. Patients’ demographic and cycle characteristics are presented in **Table 1**. The mean maternal age was 31.2 and 32.3 in the blastocyst and cleavage stage biopsy groups, respectively (p=0.05). Although no significant differences in patients’ baseline characteristics were observed between the groups, significantly higher E2 levels as well as an increased number of oocytes retrieved and fertilized (2PN) were observed in the blastocyst compared to the cleavage stage group (17,480.5 vs. 10,622, p=0.002; 24.0 vs. 18.0, p< 0.001; 15.0 vs. 11.0, p< 0.001; respectively). As expected, the number of cleavage stage HQE was significantly higher in the blastocyst compared to the cleavage stage group (12 vs. 9, p<0.001), whereas the median number of blastocysts suitable for biopsy was 8.

**Table 1:** Patient and cycle characteristics across the study groups.

Patients’ Characteristics	Cleavage stage N=64	Blastocyst stage N=83	p-value
Age (years)	32.3 ± 5.3	31.2 ± 4.1	0.05
Male partner’s age (years)	33.6 ± 5.6	34.0 ± 4.8	0.2
BMI (Kg/m^2^)	24.2 ± 5.2	23.8 ± 4.0	0.2
Smoking, N (%)	5 (8)	9 (11)	0.5
Baseline E2 (pmol/L)	149 (112 - 195)	151 (106 - 193)	0.9
Baseline FSH (IU)	7.0 (5.3 – 7.9)	6.6 (5.3 - 7.4)	0.1
Infertility, N (%)			1.0
Primary Secondary	22 (34) 42 (66)	28 (34) 55 (66)	
Cycle, oocyte and embryo parameters			
FSH dosage (IU)	2156 (1500 - 3000)	2159 (1831 - 3215.6)	0.3
No. of stimulation days, N	11 (9-12)	10 (10-12)	0.8
E2 level at triggering (pmol/L)	10,622 (8069 - 14,556.8)	17,480.5 (12,999 - 24,562.5)	0.002
No. of oocytes retrieved/OPU	18 (14 – 21)	24 (19 - 31)	<0.001
No. of fertilized oocytes (2PN)/OPU	11 (9.8 - 13)	15 (11 – 20.5)	<0.001
No. of HQE at the cleavage stage/OPU	9 (8-10)	12 (10-17)	<0.001
No. of blastocysts for biopsy/OPU		8 (5-11)	

BMI, body mass index; FSH, follicle stimulating hormone; OPU, ovum pick-up; 2PN, two pronuclei zygote; HQE, High quality embryo.

*Data is presented as mean ± SD or as median and Interquartile range (IQR).

**Figure 1:**
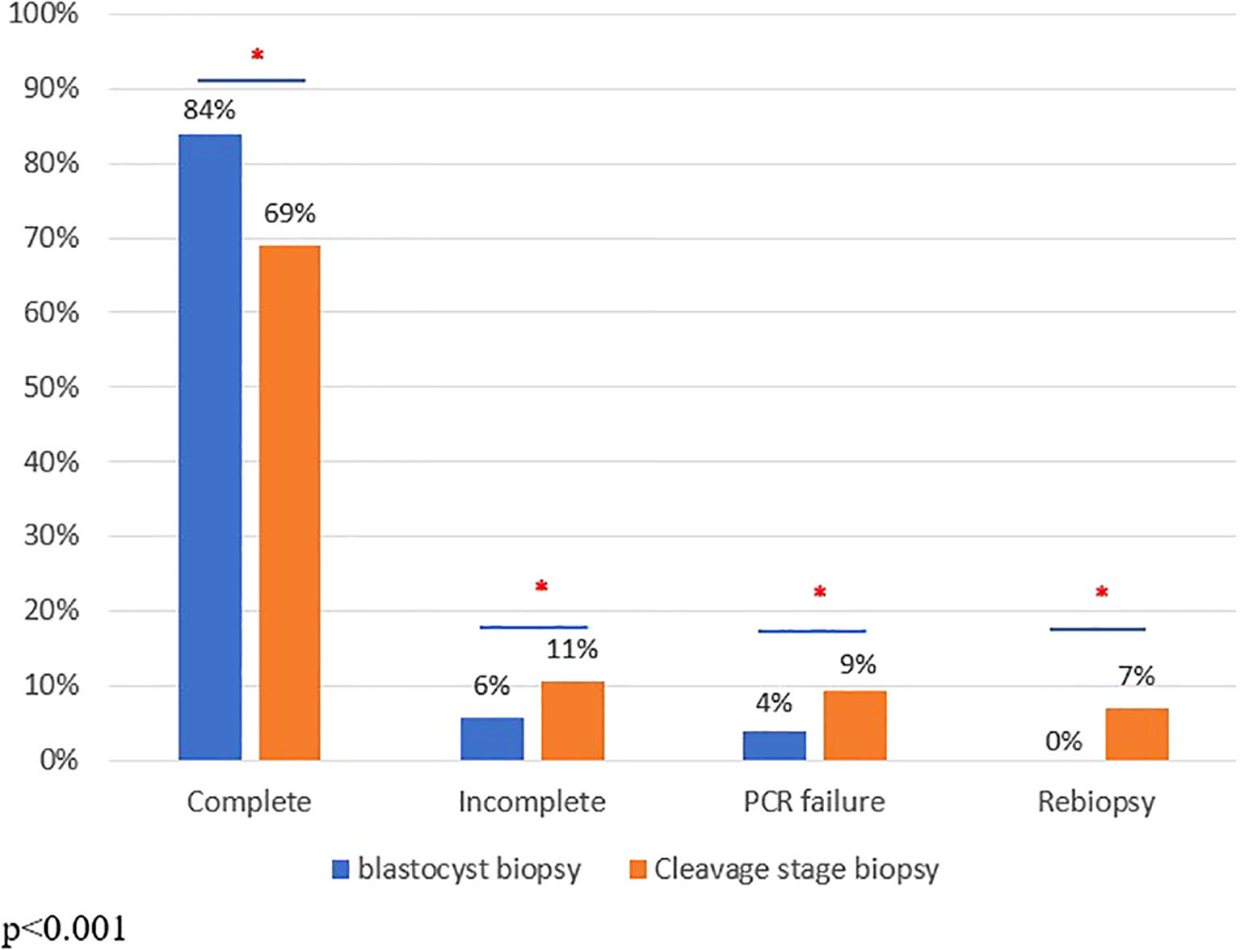
PGT-M diagnoses across the study groups. *p<0.001.

A total of 717 and 586 embryos were analyzed in the blastocyst and cleavage stage groups, respectively. In the cleavage stage group: most of the biopsies, 504, were performed at the cleavage stage while 82 were performed at the morula stage. 63 blastocysts and 147 embryos in the cleavage stage group had incomplete/PCR failure diagnosis. Re-biopsy was performed for 39 embryos in the cleavage stage group, of which 25 embryos had a complete diagnosis, while 14 embryos had incomplete/PCR failure diagnosis. The molecular diagnoses per cycle in both groups are demonstrated in **Figure 1**. Not surprisingly, a significantly higher rate of complete diagnoses was observed in the blastocyst compared to the cleavage stage group (83.6% vs 69.1%, p<0.001), while the cleavage stage group demonstrated significantly higher rates of incomplete and PCR failure diagnoses compared to the blastocyst group (10.5% vs. 5.7%, p<0.001; 9.2% vs 4%, p<0.001, respectively). Finally, a higher rate of re-biopsy was performed in the cleavage stage compared to the blastocyst group (7% vs. 0%, p<0.001).

Pregnancy outcomes are summarized in **Table 2**. A total of 121 frozen embryo transfer (FET) cycles were performed: 66 and 55 in the blastocyst and cleavage stage group, respectively. A mean of 1 and 1.1 embryos were transferred in both the blastocyst and cleavage stage groups (p=0.06), respectively, yielding no significant differences in IR (32.8%, confidence interval (CI) [23.4 -42.2] vs. 33.6%, CI [23.1 - 44.1]; p=0.9, respectively) and CPR per transfer (30.3%, CI [20.9 - 39.7] vs. 33.0%, CI [22.5 - 43.5]; p=0.7, respectively). While cumulative CPR per OPU was higher in the blastocyst compared to the cleavage stage group (46.2%, CI [35.4 – 57.0] vs. 38.3%, CI [26.3 – 50.3]), the difference was not statistically significant (p=0.4) (**Table 2A**). Of note, 10 un-affected embryos who underwent re-biopsy in the cleavage stage group were transferred, resulting in one miscarriage and one ongoing pregnancy. Further sub analysis according to the day of development of the transferred embryos between the blastocyst and the cleavage stage groups revealed no significant differences in IR (Day-5: 34.3% vs. 38.6%, p=0.7, Day-6: 15.2% vs. 7.1%, p=0.6; Day-5 vs. Day-4: 34.3% vs. 28.8%, p=0.5, respectively) and CPR per transfer (Day-5: 32.0% vs. 36.8, p=0.7, Day-6: 9.1% vs. 7.1%, p=0.6; Day-5 vs. Day-4: 32.0% vs. 28.8%, p=0.7, respectively) (**Table 2B**).

Table 2Pregnancy outcomes of the study groups – overall [A] and according to the day of development of the transferred embryos [B].

**Table:** 

A			
	Cleavage stage	Blastocyst stage	p-value
No. of embryos transferred	108	123	
No. of embryos per transfer	1.1 ± 0.3	1.0 ± 0.2	0.06
IR (%)	33.6 [23.1 - 44.1]	32.8 [23.4 - 42.2]	0.9
CPR per transfer (%)	33.0 [22.5-43.5]	30.3 [20.9-39.7]	0.7
Cumulative CPR (%)	38.3 [26.3-50.3]	46.2 [35.4-57.0]	0.4
MR (%)	7.7	11.1	0.7

IR, Implantation rate; CPR, Clinical pregnancy rate; MR, Miscarriage rate. ^*^Data is presented as means and Confidence Intervals [CI].

**Table:** 

B						
	Cleavage stage			Blastocyst stage		p-value
	**Day-4**	**Day-5**	**Day-6**	**Day-5**	**Day-6**	
No. of embryos transferred	70	29	9	93	30	
IR (%)	28.8^+^ [15-42.6]	38.6^*^ [16-60.7]	7.1^^^	34.3^*+^ [22.4-46.2]	15.2^^^	^*^p=0.7 ^^^p=0.6 ^+^p=0.5
CPR per transfer (%)	28.8^+^ [15-42.6]	36.8^*^ [14.3-59.3]	7.1^^^	32.0^*+^ [20.3-43.7]	9.1^^^	^*^p=0.7 ^^^p=0.8 ^+^p=0.7

IR, Implantation rate; CPR, Clinical pregnancy rate; MR. ^*^Data is presented as means and Confidence Intervals [CI].

Finally, no significant difference in MR was observed between the blastocyst and the cleavage stage groups (11.1% vs. 7.7%, p=0.7, respectively) (**Table 2A**).

## Discussion

The success of PGT-M cycles entails a complete genetic diagnosis of the tested embryo as well as a minimal or no detrimental effect on the embryo’s developmental and reproductive competence. Nowadays, cleavage stage biopsy is still performed in about half of the cases for PGT-M according to data analysis of 2020 presented at the last ESHRE conference, however there is an ongoing shift towards blastocyst biopsy application in recent years, with almost all of PGT-A/PGT-SR associated biopsies are performed at the blastocyst stage (5). Recent improvements in extended culture techniques and freezing methods, enabled embryo sampling at the blastocyst stage with frozen-thawed embryo transfer at subsequent cycles. In contrast to complete chromosomal testing (CCT) cycles, for which TE biopsy has become the well-established preferred method due to higher diagnostic reliability and lower mosaicism rates, leading to better pregnancy outcomes (23, 24), evidences for the purpose of PGT-M are still lacking (15). Blastocyst biopsy, involving the removal and analysis of multiple TE cells rather than a single blastomere at the cleavage stage, has been shown to reduce the rate of inconclusive diagnoses derived from allele dropout (ADO), DNA amplification failure or contamination, thus improves the diagnostic sensitivity of PGT-M (24, 25). Our results are in agreement with those of previous studies and the recent published data of the ESHRE consortium demonstrating higher rate of complete diagnoses as well as lower rates of incomplete and PCR failure diagnoses while performing blastocyst compared to cleavage stage biopsy (Complete:83.6% vs 69.1%; Incomplete:5.7% vs. 10.5%; PCR failure: 4% vs 9.2%, p<0.001for all, respectively) (25). Although in our study all the embryos in the cleavage stage group which underwent re-biopsy survived the procedure, eventually a higher rate of embryos were “lost” in the cleavage stage compared to the blastocyst stage group (20.8%, 122/586 vs 8.7%, 63/717, respectively) due to inconclusive genetic results. Given the aforementioned, it may therefore be suggested that blastocyst biopsy should be the preferred method due to higher diagnostic efficiency. Nevertheless, not every embryo would reach the blastocyst stage of development, hence deferring the biopsy and extending the culture beyond the cleavage stage, in several patient populations, might result in a grim outcome of no embryos for testing and transfer. Our group has recently demonstrated that the mechanism responsible for embryos arrest *in vitro*, between the cleavage and blastocyst stages, is not embryo aneuploidy, but rather other, such as culture conditions, suggesting that blastocyst transfer might lead to the loss of embryos that may have survived *in vivo* (11). This was recently confirmed by Xiao et al. who demonstrated increased pregnancy and live birth rates per OPU following cleavage stage compared to blastocyst transfer, when only one embryo developed, implying that the uterus might be a better incubator than the laboratory (14). Considering the latter, an evaluation of cumulative pregnancy rate per initiated cycle between the two biopsy strategies remains highly relevant and is the only outcome that takes into account the cancelled transfer cycles. Importantly, a significantly higher rate of re-biopsy was performed in the cleavage stage compared to the blastocyst group (7% vs. 0%, p<0.001). Although the number of re-biopsies has a non-negligible effect on the clinic’s laboratory workload as well as the cycle’s costs, the extent of those varies according to the number of embryos available for biopsy at the cleavage stage in specific patient populations (hyper versus poor responders) and should be weighed against the possibility of having no embryos for testing and transfer, especially in the poor responder patient population. Additionally, the main objective of the current study, focuses on the reproductive outcomes of the two biopsy strategies, for which the evidence is insufficient to support the superiority of one strategy over the other, as was concluded in the recent Cochrane review (15).

One of the main concerns that was raised in regard to PGT cycles relates to a possible negative effect of the biopsy itself on the embryo’s reproductive potential. It has been suggested, according to *in-vitro* studies, that cleavage stage biopsy involving the removal of a larger portion of the embryo as well as cells that will contribute to the embryo proper compared to blastocyst biopsy, might have a detrimental effect on embryonic development. While two earlier studies demonstrated delayed post cleavage stage biopsy morphokinetic parameters up to the blastocyst stage (26, 27), a larger recent study contradicts these observations showing earlier onset of these parameters in biopsied compared to unbiopsied cleavage stage embryos (28). The mechanism underling these modifications still remains unknown and given this incongruency, further evaluation of post biopsy *in-vivo* competence, as presented in our study, is of utmost importance. The current study is the largest, so far, comparing the reproductive outcomes between cleavage and blastocyst biopsy in PGT-M cycles. In our study, 98.4% and 87.2% of un-affected transferrable cleavage stage embryos survived to Day-4 and the blastocyst stage, respectively. Our observations are in concordance to the survival rate of good quality Day 3 un-biopsied embryos to the blastocyst stage as was previously described (29). Furthermore, we evaluated the outcomes of cycles with at least eight high quality embryos at the cleavage stage that were subjected to freeze-all in both groups to avoid a possible effect related to the type of transfer cycle (fresh vs. frozen) on the pregnancy outcomes, as was previously demonstrated in hyper-responders (30). Our findings reveal comparable IR, CPR per transfer and cumulative CPR per OPU between cleavage stage and blastocyst biopsy strategies. Previous studies evaluating these outcomes between the two strategies are scarce. A milestone study by Scott et al. concluded that cleavage stage biopsy markedly reduced embryonic implantation potential compared to trophectoderm biopsy (8), However, a careful inspection of this study reveals an incredibly high sustained implantation rates of the unbiopsied cleavage stage embryos (50%) which were comparable to those of the biopsied and unbiopsied blastocyst stage embryos (54% and 51%, respectively). This observation is in contrast to well-established evidence, as presented in the recent Cochrane review, demonstrating higher clinical pregnancy and live birth rates following fresh blastocyst compared to cleavage stage transfer (31). Another older study by Kokkali et al. also reported of lower implantation rates when a cleavage stage biopsy plus blastocyst stage transfer workflow was adopted, rather than a blastocyst stage biopsy and a fresh transfer (27% vs. 48%, respectively, p=0.1) (16). However, this study included a small sample size (N=10 patients in each group), the difference in implantation rates did not reach statistical significance and cumulative pregnancy rate per treatment cycle wasn’t evaluated. Moreover, calculating the rate of pregnancies reaching term per cycle (40%) fails to show difference between the groups, leading to the conclusion that cleavage stage biopsy may not be inferior to blastocyst biopsy for PGT-M purposes in regards to clinical outcomes. Our observations of 30.3% and 33% clinical pregnancy rates per transfer in the blastocyst and cleavage stage biopsy groups respectively are also supported by similar rates (35%), as reported in the recent ESHRE consortium data analysis of PGT-M cycles from 2016 to 2017 (25). These comparable figures may be explained by the fact that embryos undergoing cleavage stage biopsy are transferred a day or 2 later, at the morula or blastocyst stages. As was previously described, in our IVF unit, frozen biopsied cleavage stage embryos that were found un-affected are thawed at subsequent cycles, cultured overnight and are transferred at the morula or blastocyst stage. This practice enables us to assure the transfer of embryos that showed post-thawing developmental competence. Studies in preimplantation human embryos suggest that the compaction process could be an important checkpoint for embryo quality since it involves a self-correction mechanism in which aneuploid cells are expelled from the embryo (32, 33), thus provides another timepoint, post cleavage stage, during which embryo selection occurs. The lack of significant difference in IR and CPR per transfer between the groups, in our study, could be explained by our transfer practice, as previous studies in non-biopsied embryos demonstrated comparable IR and CPR in elective single embryo transfers (eSETs) at the morula and blastocyst stages (34, 35). The aforementioned evidence also supports the observation of no significant difference in MR between the groups in our study. Given the lack of evidence for a detrimental effect of blastomere compared to TE biopsy on the embryonic reproductive competence, as shown in our study, as well as comparable pregnancy outcomes per transfer of morula vs. blastocyst, the possible advantage of cleavage stage biopsy is an earlier transfer of the embryo to its natural environment, the uterus. Previous studies have shown increased incidence of monozygotic twinning as well as altered sex ratio in favor of males related to blastocyst culture (36–38). Monozygotic twins (MZT) pregnancies bear higher incidence of maternal and perinatal morbidity (gestational diabetes, preeclampsia, low birth weight, twin to twin transfusion syndrome, intrauterine fetal death etc.) thus increases the overall risks associated with pregnancies derived from blastocyst transfers. Whether the underlying mechanisms leading to these modifications relate to altered epigenetic gene expression inflicted by the media constituents (39),delayed implantation (40) or a morphological selection criteria used for decision upon extended culture (41, 42), need to be elucidated. Furthermore, recent large studies reported of an association between TE biopsy and increased incidence of hypertensive disorders of pregnancy (43, 44). This association remained significant even when comparing between pregnancies derived from blastocyst biopsy to those derived from cleavage stage biopsy (45). These negative outcomes should also be taken into consideration in the decision of the timing of the embryo biopsy as well as transfer. Lastly, Performing the biopsy at the cleavage stage also provides the opportunity to perform a fresh embryo transfer. While there is no evidence of a significant difference in effectiveness, measured by live birth and ongoing pregnancy rates, between the freeze-only and the fresh ET strategies (46), the incremental cost of freeze-only compared with a fresh ET for a 1% additional live birth rate is very high (47), another factor that needs to be taken into account when deciding upon the treatment strategy. In our study, freeze-all was performed to reduce the risk for ovarian hyperstimulation syndrome (OHSS) in our study population of hyper-responders, allowing us to evaluate the effect of the biopsy itself on the reproductive outcomes. Studies including normal/poor responders are needed to evaluate the possible benefit of cleavage stage biopsy and fresh ET compared to blastocyst biopsy and freeze-only strategy.

A major strength of the current study is the solely inclusion of cycles that were subjected to freeze-all in good prognosis patients, hence neutralizing the possible effect of the type of transfer cycle on the pregnancies’ outcomes.

There are several limitations that also need to be considered. First, our data represent the results from a single fertility center which might limit the generalizability of our results. Nevertheless, it could also be considered as a strength due to uniformity of the stimulation protocols employed, the homogeneity of the population as well as the methods for embryo biopsy and genetic analysis performed by a single center laboratory. Second, while the findings of this study are valuable and shed light on the association between the timing of the biopsy and the pregnancy outcomes, due to its retrospective nature it was impossible to control for all the parameters directing the staff’s decision towards cleavage rather than blastocyst biopsy and vice versa. Third, the small number of Day 6 embryos in both groups impairs our ability to draw conclusions regarding the effect of the timing of the biopsy on those embryos’ reproductive outcomes. However, it is reasonable to assume that the general clinical policy wouldn’t be dictated by the results of this small group.

In conclusion, the implantation potential following blastomere biopsy is not compromised as compared to trophectoderm biopsy. Moreover, no significant difference in pregnancy outcomes is observed between the two biopsy strategies in good prognosis patient population undergoing freeze-all cycles. Blastocyst biopsy shows higher efficiency compared to cleavage stage biopsy as evident by a higher rate of complete diagnoses and lower rates of incomplete/PCR failure results, thus reduces the need for re-biopsy. Our findings could assist in clinical decision making upon the preferred timing of the embryo biopsy according to patients’ characteristics. In patients undergoing PGT-M cycles having at least eight high quality embryos suitable for biopsy at the cleavage stage, performance of blastocyst over cleavage stage biopsy should be considered due to higher cost-effectiveness. Nonetheless, TE biopsy and blastocyst transfer related morbidities should also be accounted for, especially in patients at risk for pregnancy induced hypertensive disorders. In patients with less than eight high quality embryos at the cleavage stage, especially in the poor prognosis patient population, having only few embryos at the cleavage stage, performance of cleavage stage biopsy and a fresh embryo transfer can be considered, without the concern of compromising the reproductive outcomes. Additional larger studies including different patient populations, evaluating cumulative live birth rates per initiated cycle performing blastocyst vs. cleavage stage biopsy are warranted to substantiate our results as well as to enable an optimal tailored treatment to the PGT-M patient population.

## Data Availability

The raw data supporting the conclusions of this article will be made available by the authors, without undue reservation.
